# A new screening framework to support the identification of exogenous particles and suspect microplastics *in situ* in pathological tissue samples

**DOI:** 10.1016/j.ebiom.2025.105984

**Published:** 2025-11-03

**Authors:** Stephanie L. Wright, Claire Gwinnett, Ian S. Mudway, Yukari Ishikawa, Henry Blake, James M. Kinross, Frank J. Kelly, Jon Salisbury

**Affiliations:** a Environmental Research Group, School of Public Health, Imperial College London, London, United Kingdom; b Medical Research Council Centre for Environment and Health, School of Public Health, Imperial College London, London, United Kingdom; c Criminal Justice and Forensic Science Department, School of Law, Policing and Forensic Science, Staffordshire University, Stoke-on-Trent, United Kingdom; d Department of Surgery & Cancer, Imperial College London, London, United Kingdom; e Department of Histopathology, King's College Hospital, London, United Kingdom

**Keywords:** Particles, Microplastics, Pathology, Methodological framework, Gastrointestinal tract, Polarised light microscopy

## Abstract

**Background:**

Microplastics are ubiquitous environmental pollutants that have been detected in various human tissues. Often the particle sizes reported challenge established theory in toxicokinetics, and fundamental data on whether exogenous microparticles can enter tissues (and which tissues) is needed. This observational and method validation study aimed to develop a screening framework to determine whether particles including microplastics can access tissues using human ileal tissue sections and to investigate the presence of exogenous particles within the subepithelial mucosa.

**Methods:**

As proof of concept that exogenous particles may translocate into tissues, a screening framework was established using polarised light microscopy (PLM), to identify birefringent particles in ileal tissue sections from 101 subjects. The population included adults who had undergone endoscopy and biopsy for the investigation of bowel symptoms at King's College Hospital. Exclusion criteria were applied to differentiate between true particles and procedural contaminants. The remaining particles were analysed for their morphology and size. To further illustrate the potential for plastic to access intestinal tissue, a bulk intestinal tissue sample underwent a basic digestion, filtration, and analysis using pyrolysis gas chromatography mass spectrometry.

**Findings:**

Results revealed the presence of subepithelial birefringent particles in a small number of ileal tissue sections. However, of the 35 samples containing sub-epithelial particles, 31 (i.e., 91% of observations) were concluded to be due to procedural contamination. Of the ‘true’ observations, some of these particles, which include starch, likely entered the tissue via persorption. Additionally, a single viscose fibre was observed within the lumen of the small intestine. Supplemental chemical data finding signatures of polypropylene and polyvinylchloride demonstrate how the framework would fit in a wider analytical pipeline.

**Interpretation:**

This study highlights the importance of a robust screening framework for the identification of particles and suspect microplastics in human tissues. While the frequency of particles like microplastics in the subepithelial mucosa was low, the findings suggest that microplastics could indeed penetrate the intestinal epithelium and this was supplemented with example chemical data. Further research is needed to investigate the distribution, accumulation, and potential health effects of microplastics in human tissues.

**Funding:**

10.13039/501100000265Medical Research Council.

**Research in context**

**Evidence before this study**

Populations are exposed to a variety of exogenous particles, including microplastics. Microplastics have been detected in a variety of human tissue samples, although the sizes of these particles often contradict established theories of particle biokinetics. Furthermore, most studies analyse digested bulk tissue, removing any biological context with respect to tissue localisation. Pathological tissue sections provide a valuable resource from which to explore whether exogenous particles, such as microplastics, can access specific anatomical regions or are linked with pathologies, prior to more in-depth characterisation studies.

**Added value of this study**

By employing a rigorous suspect screening framework, including expert histopathological input, we have determined that a significant proportion of exogenous particles observed in tissue sections are likely procedural contaminants. This finding carries implications for past, present, and future studies investigating microplastics or other solid particles in pathological tissue sections. We have also observed large microparticles in the gut mucosa, suggesting that microplastics could indeed penetrate the intestinal epithelium. Considering the rapid growth of microplastic research specifically, especially in clinical settings, we believe that this knowledge will enhance the scientific rigour of future studies.

**Implications of all the available evidence**

Collectively, these findings aim to highlight critical issues and promote the adoption of robust research practices when investigating pathological tissue sections for exogenous particles, including microplastics, which is gaining significant attention in the medical community. This foundational work informs a broader research pipeline, demonstrating the presence of particles as a prerequisite for more detailed characterisation. We believe that this will contribute to the accuracy and reliability of future studies and ultimately deepen our understanding of the potential health effects of microplastic exposure.

## Introduction

Through pollution, additives and nano-enabled products, and some occupations such as construction, mining, and agriculture, humans are exposed to a variety of micro- and nanosized particles daily. Some source-specific particle/fibre exposures, such as traffic-related air pollution,^1^ asbestos,^2^ and wood smoke,^3^ are known to drive adverse pathophysiology. An important step in establishing the potential hazard, and ultimately the risk of any particulate exposure to human health, requires a comprehensive understanding of their absorption, distribution, metabolism, and excretion (ADME) behaviour *in vivo*, prior to an evaluation of whether the tissue doses are sufficient to induce adverse biological responses.^4^^,^^5^ Given the recent interest in microplastics amongst the particle and fibre toxicology, clinical, and epidemiological research communities, this particle class will form the focus of this paper, although the principles are applicable to any tissue-borne particle.

Microplastics encompass a heterogenous mixture of particles spanning different shapes, and degradation states across several orders of magnitude in size, originating from a range of commonly used plastic polymer-based materials.^6^ Humans are largely exposed to microplastics via ingestion and inhalation routes. This is supported by numerous studies reporting on the presence of microplastic in different environmental and dietary media (reviewed by^7^). The first point of contact between any particle inhaled in air, or consumed within food stuffs, are the mucosal membranes of the lung and gut, respectively.^8^ These barriers are complex environments of extracellular fluids, including mucus, overlaying structural and immune cells that function to exclude foreign material from being taken up into the body, across the exposed epithelium (gastrointestinal or airway) to reach the circulation prior to distribution to other organs.

To evaluate microplastic uptake into the body, numerous groups have examined specimens of different human organs to determine the presence of microplastic particles (including fibres), to demonstrate that absorption, distribution, and potentially accumulation has occurred. Publications have reported on the discovery of microplastic particles in a range of digested human tissues, including distal organs such as placenta,^9^ liver,^10^ and testes.^11^ This indicates that both absorption across an epithelial barrier and subsequent distribution, either via the vasculature or lymphatic system, has occurred. However, the particle sizes reported in these tissues (often in excess of 20 μm) and their distribution throughout the body challenge established theory in particle biokinetics,^7^ where restrictions are placed on which particle sizes can cross epithelial barriers and cell walls or be transported via capillary and lymphatic networks.^12^ Additionally, without the context of the surrounding tissue structure, whether the particles are truly incorporated in tissue, i.e., intra-cellularly or interstitially, are mobilised in biofluids (blood or lymph), or are an artefact due to procedural contamination cannot be determined. For accumulation to occur, there must be incorporation into cells and/or tissues. Distinguishing where this happens is important as it represents a site where the dose is likely to increase over a chronic exposure, potentially to harmful levels, and thus highlights sensitive target organs or systems. Furthermore, discriminating artefacts is paramount, as the contemporary laboratory environment is subject to high levels of background microplastic contamination^13^ and given the ethical issues to consider when reporting on findings with implications to human health, the level of confidence in the data must be unequivocal.^14^

Pathological tissue archives present a resource from which to identify exogenous particles *in situ* in the context of the surrounding tissue, indicating internal exposure and through correlation with histopathological features and biomarkers, their relation to adverse responses.^15^ However, there are few published studies on microplastic particles in intact human tissue *in situ*.^16^ Before the onset of resource intensive research adopting both imaging and chemical analysis of particles in human bulk tissue or sections, a priori evidence regarding whether exogenous particles are at all observed beyond the epithelial point of entry is needed as justification for further work.

As mentioned previously, both the airway and gastrointestinal epithelium are considered points of entry for particles due to their exposure to the external environment. Of the two, larger particles (>1 μm) are known to cross the GI mucosa.^17^ Chemical analysis to confirm the composition of particles of this size is theoretically possible with microspectroscopy without the need for further advanced techniques, as would be required for sub-micron particles. Regions of the gut—the Peyer's patches—host M-cells, which specialise in particle uptake, whilst persorption (the mechanical translocation of non-digestible particles up to 130 μm across the epithelium) can also occur along the small intestine.^18^ If microplastic uptake across the gut were to occur, then the ileum would represent an ideal target tissue to begin assessment. Here we establish a methodology for investigating particle and fibre penetration into tissues and present an exemplar study using human ileum pathological samples. This initial ‘suspect screening’—demonstrating the presence of exogenous particles, which could include microplastics, but also other particle types, is essential in justifying more targeted, time and resource intensive research.

Within any analytical pipeline, an initial screen using a relatively quick technique would clearly be advantageous in building evidence of the presence of subepithelial microparticles to justify the need for more time intensive research including chemical analysis of bulk tissue via pyrolysis gas chromatography mass spectrometry (Py-GCMS) or tissue sections via vibrational microspectroscopy. Polarised light microscopy (PLM) has been used for more than seventy years to identify plastic particles in histological specimens,^19^ where strongly birefringent particles were likely shed from prosthetic implants. Whilst difficult to discern whether birefringent particles are microplastics in the absence of a clear source, the technique presents a first step in identifying exogenous particulate matter and could be adopted as an initial screening step: a) to provide evidence supporting the need for further research, and b) for reducing the sample number for more time-intensive analytical techniques.

Quality Assurance/Quality Control (QAQC) frameworks are in place for microplastic analysis techniques (e.g.,^20^), however, none have been prescribed for analysing microplastic directly in tissue sections and thus there is still a need for these to be developed. The combination of both a screening framework for assessing whether observed particles and microplastic are true positives (or artefacts) and preliminary evidence on microparticle absorption in epithelial tissues will pave the way for robust microplastic research *in situ* in human tissues. The current study therefore proposes and applies a screening framework to increase the level of confidence in *in situ* particle and microplastic data from pathological tissue sections. Additional to this, it acts as a proof of principal in determining whether there are discriminate birefringent foreign particles embedded in ileal tissue. Thus, this work should be viewed as a suspect screening method for particles and fibres in tissue mucosa, serving as a foundation for designing and justifying future studies that incorporate chemical analysis. This foundational work informs a broader research pipeline, demonstrating the presence of particles as a prerequisite for more detailed characterisation. This is timely as publications and research on microplastic biomonitoring continues to increase (e.g.,^16^^,^^21^^,^^22^), often with significant debate about the authenticity of identification.^23^ With the increased media interest in the topic the field needs to ensure that headline findings do not lose sight of fundamental metrological principles, pathological context and plausibility required to inform evidence-based policy in this area.

## Methods

### Study design

To achieve the aim of designing and applying a screening framework to increase the level of confidence in *in situ* particle and microplastic data from pathological tissue sections, archived Haematoxylin and Eosin (H and E) ileum tissue sections were used and screened. The findings act as a proof of principal in whether discriminate (birefringent) foreign particles gain access to this tissue and the framework can be applied to other tissues more widely. Whilst not the focus, to merely demonstrate how this would proceed to future research should the appropriate tissue be available/procured, an example colon tissue sample underwent processing and chemical analysis.

### Ethics

Ileal tissue: The study was approved by the Research Ethics Committee and NHS Health Research Authority and sponsored by King's College Hospital NHS Foundation Trust (18/EE/0186, 2018). The ethics did not cover demographic information, as the study was about proof of concept that particles gain access to tissues using a developed screening framework. Written informed consent had been obtained from all patients for the original diagnostic biopsies. The hospital ethics committee deemed that written informed consent not necessary for this specific research because the samples were anonymised.

Colon tissue: The colon tissue used in this research project were obtained from the Imperial College Healthcare Tissue Bank (ICHTB). ICHTB is supported by the National Institute for Health Research (NIHR) Biomedical Research Centre based at Imperial College Healthcare NHS Trust and Imperial College London. ICHTB is approved by Wales REC3 to release human material for research (17/WA/0161), and the samples for this project (R21058) were issued from sub-collection reference number 17/WA/0161.

### Tissue procurement

Ileal tissue: The screening framework was developed and validated on ileal tissue that had already been taken for diagnostic purposes. Ileal (small intestine) mucosal samples were obtained from 101 adult patients who had undergone endoscopy and biopsy for the investigation of bowel symptoms at King's College Hospital, London. All biopsies had been fixed in 10% formal saline and embedded in paraffin wax. Three-micron thick sections were cut at three different levels through each biopsy (30 μm between each level) and stained with haematoxylin and eosin for light microscopy. Each biopsy was reported previously by a consultant gastrointestinal histopathologist as showing normal morphology with no evidence of disease and was anonymised prior to being used for the study. No other information was accessed and used for the purpose of this study.

Colon tissue: An exemplary colon tissue sample (large intestine) was analysed for its plastic content as particle uptake can happen across this region.^24^ This specimen was obtained prospectively during routine excision of bowel surgery for colon and rectal cancer. The fresh colonic sample was opened in the operating room and sampled using a sterilised steel blade, 10 cm proximal and distal to the target lesion and from the cancer or polyp itself. The specimen was carefully transferred to an aluminium specimen jar with an aluminium screw cap, which had undergone the pre-cleaning procedure (see QA/QC Statement below), and was stored at −80 °C until analysis.

### *In situ* particle screening framework (ileal tissue)

A maximum of three slides per subject were examined, depending on how many contained ileal tissue, which was identified by the presence of villi and lymphoid aggregations (Peyer's patches). The **first tier** in the framework consists of an optical microscopy screen using a mode of microscopy which maximises the contrast of potential microplastic and exogenous particles from tissue. In this instance, we used PLM to screen for the presence/absence of birefringent particles. Screening was performed by orientating the slides with their sample label to the right under an Olympus BX51 microscope using 40× magnification and bright-field to navigate across sections. Once an ileal section was intercepted, the whole section was Z-screened at 200× magnification under crossed-polar conditions with polarised light for the presence of birefringent particles.

The **second tier** of the framework was to further screen these sections with subepithelial birefringent particles according to exclusion criteria. The tissue surrounding birefringent particles was inspected at 400× magnification, switching between polarised light and crossed polar conditions. The screening resolution with this set-up was limited by what is distinguishable by eye at this magnification (1–2 μm) during the screen. Particles were excluded based on the following criteria: 1) proximity to clusters of red blood cells, indicative or trauma during sampling, as the particle may have been introduced into the tissue as an artefact; 2) proximity to the base of the tissue section, as the particle may be an artefact of contaminated adhesive tape on which the tissue was placed; 3) presence of tissue overlaid on the particle, as this may be an artefact of a contaminated slide on which the section was mounted; 4) distorted tissue/cell structures surrounding the particle, as this implies forced intrusion, likely an artefact of the sampling process/preparation; and 5) the particle being in a different focal plane to the tissue section, as this also implies procedural contamination by being deposited on top of the section rather than being within.

After applying exclusion criteria, remaining tissue sections containing birefringent particles were imaged using an Olympus BX51 microscope with an Olympus UC30 camera (resolution 3.2 megapixels) and Olympus Standard CellSens software program (Version: Standard 1.16, build no. 15404, copyright 2009–2016) for further scrutiny by a pathologist, **tier 3** of the framework. The pathologist further distinguished tissue sections containing birefringent particles which could not be confidently attributed to procedural contamination as per the exclusion criteria and were therefore considered an observation (included). The subepithelial birefringent particles in the remaining tissue sections were approximately sized using Image J software 1.52j.^25^

### Fibre identification

A tissue section from one subject contained a suspicious birefringent fibre in the lumen. The observed microfibre was further examined under two high powered microscopes; the optical properties (birefringence and sign of elongation) of the fibre were analysed under a binocular James Swift polarising light microscope at 400× magnification and the width and thickness measurements (for birefringence calculation) were measured at 400× magnification using a calibrated Olympus BX51 microscope with an Olympus UC30 camera and Olympus Standard CellSens software program (as above). Five width measurements were taken along the length of the fibre including both ends. The presence of any inclusions and the cross-sectional shape of the fibre were also observed under both microscopes. The Optical Path Difference (OPD), also known as retardation, was identified using a James Swift quartz wedge and Michel Levy chart (University of Liverpool printed version).^26^

### Chemical analysis (colon tissue)

Should the above screening framework establish the presence of particles and fibres in tissues, this would provide justification for the design and conduct of futures studies that incorporate chemical analysis in a step towards identification. Thus, the above framework informs a broader research pipeline, demonstrating the presence of particles as a prerequisite for more detailed characterisation. To further illustrate the pipeline's functionality, representative Py-GCMS data demonstrating the presence of plastic polymers in digested bulk gut tissue has been included. This strengthens the argument for the utility of this tiered approach, enabling researchers to prioritise samples and justify more resource-intensive chemical analyses. This is detailed in the following subsections.

### QAQC statement for chemical analysis

To minimise background microplastic contamination from the sampling, storage, and laboratory procedures, only stainless steel, aluminium and glass items were used. All items underwent a rigorous washing procedure. First, they were washed with washing detergent, then rinsed with running tap water and soaked in alkaline laboratory detergent overnight. The next day, items were rinsed thoroughly with running tap water, rinsed with MilliQ water and then dried in an enclosed temperature controlled drying cabinet dedicated to microplastic analysis workflows. Glass fibre filters (Whatman GF/F, 47 and 25 mm, 0.7 μm) were first conditioned in a muffle furnace at 600 °C for 1 h. All reagents used (KOH, EtOH etc) were filtered to 0.7 μm, the same pore size as samples were filtered to. During sample collection, the operating theatre was previously an orthopaedic theatre, and thus had a HEPA filtered air system. Sample preparation was conducted in an ISO Class 5 clean hood within an ISO Class 8 clean laboratory dedicated to microplastic research. Clean room protocols were followed in relation to putting on PPE and conducting work within the environment. Operators wore a no-shed laboratory coat, and clean room gloves. Any activity conducted in the main laboratory (incubations, freeze-drying) were done using covered samples or within enclosed environments. A procedural blank which underwent the whole process without a sample was processed and analysed in the same way.

### Sample preparation for pyrolysis-GCxGC-TOFMS

The colon tissue sample was allowed to thaw in its container in the clean lab. In the clean hood, the tissue sample was transferred to a new pre-weighed glass sample jar using stainless-steel forceps. It was roughly cut within the jar, into as many pieces as possible using stainless-steel scissors and the jar opening was covered with aluminium foil. The jar was re-weighed to derive a sample mass, carefully lifting the foil slightly above so as not to interfere with the weight. The sample was then transferred to a freeze drier and dried for 24 h. After 24 h, the sample was left until it reached room temperature. Any condensation on the outside of the jar was carefully wiped using lint-free tissue paper, and then the jar was re-weighed again.

Having recorded a dry weight, the sample was transferred into a 100 mL wide-neck Erlenmeyer flask using stainless steel forceps and a 5M KOH solution was added at 10:1 volume: weight. If any tissue remained in the jar, a small volume of MilliQ water was used to rinse it into the Erlenmeyer flask. A cleaned Petri dish was placed on top of the Erlenmeyer flask for avoiding the background contamination. The sample was placed on an orbital shaker (speed level 6, room temperature) for 48 h to allow digestion to take place.

After 48 h, 17.5 mL of EtOH was added to each Erlenmeyer flask^27^ in the clean hood in the clean laboratory. Then, the sample was covered and left on the orbital shaker for a further 24 h. Finally, the digested sample was filtered onto a 25 mm filter in a glass filtration unit. The filter was transferred to a glass Petri dish using clean forceps, which was covered with its lid and dried at 40 °C for 24 h. A weight for the dried filter was taken, then the filter was subsampled using a metal biopsy punch (2 mm). Seven punches were taken in an array—a transect across the diameter, and a perpendicular transect across the radius, from the sample edge. These filter punches were placed in an OPTIC-4 CDCS liner (GL Science, Japan) and were capped ready for analysis.

### Pyrolysis-GCxGC-TOFMS

Qualification of the presence of target plastic analytes in colon tissue was performed using an Agilent 7890B GC with a LECO Pegasus BT 4D Time of Flight Mass Spectrometer detector fitted with a PAL-3 autosampler (Agilent) and Optic-4 multimode inlet (GL sciences). The sample underwent both thermal desorption by heating to 300 °C (to clean up the sample further by removing potential volatile interferences) and pyrolysis heating to 600 °C to thermally decompose any non-volatile plastic polymers remaining in the sample. The pyrolysis products were separated and detected. Details of the instrument conditions are in Table 1.

**Table 1:** Analytical conditions for the analysis of plastic polymers in digested colon using pyrolysis- GCxGC-TOFMS.

Pyrolysis conditions	First shot temperature	300 °C
	Second shot temperature	550 °C
	Transfer line temperature	300 °C
	Pyrolysis time	60 s
GC conditions	Model	Agilent 7890
	Primary column	HP-5MS: 30M × 0.25 mm
	Secondary column	Rxi-17 ms: 1m × 0.25 mm
	Primary oven programme	50 °C [2 min hold] →10 °C Min^−1^ → 250 °C → 20 °C Min^−1^ → 300 °C [15 min hold]
	Secondary oven programme	55 °C [2 min hold] →10 °C Min^−1^ → 255 °C → 20 °C Min^−1^ → 305 °C [15 min hold]
	Split ratio	200:1
	Carrier gas	Helium
MS conditions	Ion source temperature	250 °C
	Ionisation energy	70.0
	Scan range	40–600 mu
	Emission current	1.0 mA
	Acquisition rate	250 scans s^−1^

A microplastics standard in CaCO_3_ diluent, which contains 12 common plastic polymers (Frontier Laboratories Ltd., Fukushima, Japan), was used to aid qualification. The standard powder was added into a muffled DMI insert and analysed. All data was processed using ChromaTOF software 4.30 by building a targeted analyte finding method based on the marker ions of the target polymers, with a match threshold >800 for markers (Table 2). Identified peaks were also manually checked for their peak shape and mass spectra.

**Table 2:** The pyrolysis products targeted for the 12 plastic polymers.

Polymer	Pyrolysis products	*m*/*z*	Retention Time
Polyethylene	Alkane, Alkene, Alkadiene (C10–C21)	Numerous	Numerous
Polypropylene	2,4-dimethyl hept-1-ene	43, 70, 126	220–250
Polystyrene	Dimer	91, 208	1000–1070
	Trimer	91, 312	1370–1455
Acrylonitrile butadiene styrene	SAS	91, 170, 261	1316–1334
	SSA	144, 260	1304–1313
	ASS	91, 117, 261	1277–1283
Styrene butadiene Rubber	4-phenylcyclohexene	104, 158	710–730
	4-vinylcyclohexene	54, 79, 108	210–250
Polymethyl methacrylate	Methyl Methacrylate	69, 100	135–150
Polycarbonate	Bisphenol A	213, 228	1315–1390
Polyvinyl chloride	Indene	115, 116	425–460
	Naphthalene	128	575–600
	Fluorene	82, 166	920–950
Polyurethane	4,4′-Methylenedianiline	106, 198	1300 = 1340
Polyethylene terephthalate	Biphenyl	76, 154	750–780
Nylon-6	Caprolactam	84, 85, 113	625–700
Nylon-6,6	Cyclopentanone	55, 84	185–210

### Statistics

*In situ* Particle Screening Framework (ileal tissue): Due to a low number of observations, i.e., four particles across three (out of 101) subjects, statistics was not deemed necessary. Sample size (at least n = 100 subjects) was nominally chosen. The inclusion criteria was ileal tissue conforming to normal morphology with no evidence of disease. The exclusion criterion was other anatomical regions, such as colon.

Chemical analysis (colon tissue): This was performed on an exemplary sample, to demonstrate the integration of the framework in a wider analytical pipeline and was not the intended focus of the study. The inclusion criteria were adult patients (age 18 years or over) undergoing elective surgery for colorectal cancer, who were able to give informed consent and were neo-adjuvant treatment naive. Exclusion criteria included emergency surgery, previous stoma, age under 18 or unable to consent.

### Role of funders

The funders of the study had no role in study design, data collection, data analysis, data interpretation, or writing of the report. The authors had full access to the data in the study and accept responsibility for the decision to submit for publication.

## Results

### *In Situ* particle screening framework

**Fig. 1:**
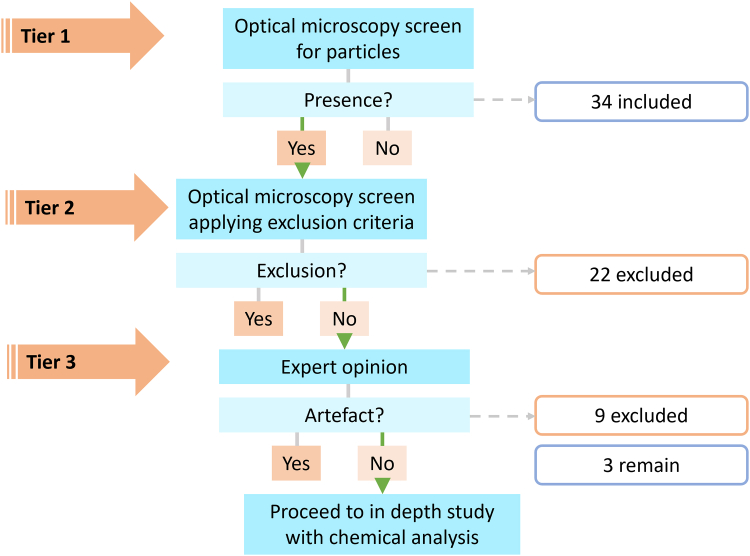
A schematic showing how many samples were excluded at each tier of the *in situ* Particle Screening Framework in this case study. Slides including H&E histopathology sections from over 100 subjects were screened.

**Fig. 2:**
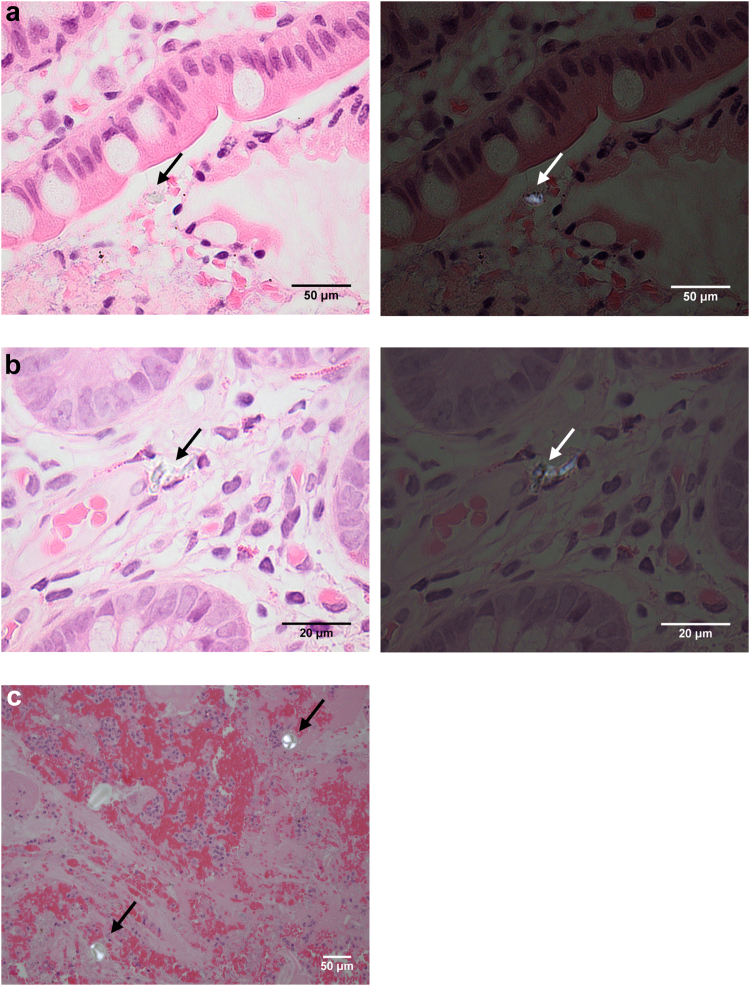
Micrographs of the resulting ileum tissue sections after applying the 3-tier *in situ* Particle Framework to 101 cases and their corresponding crossed polar-polarised light micrograph. Arrows indicate either suspicious subepithelial birefringent particles (a and b) or suspected starch particles (c). Scale bars = 50, 20 and 50 μm for a, b and c, respectively.

**Fig. 3:**
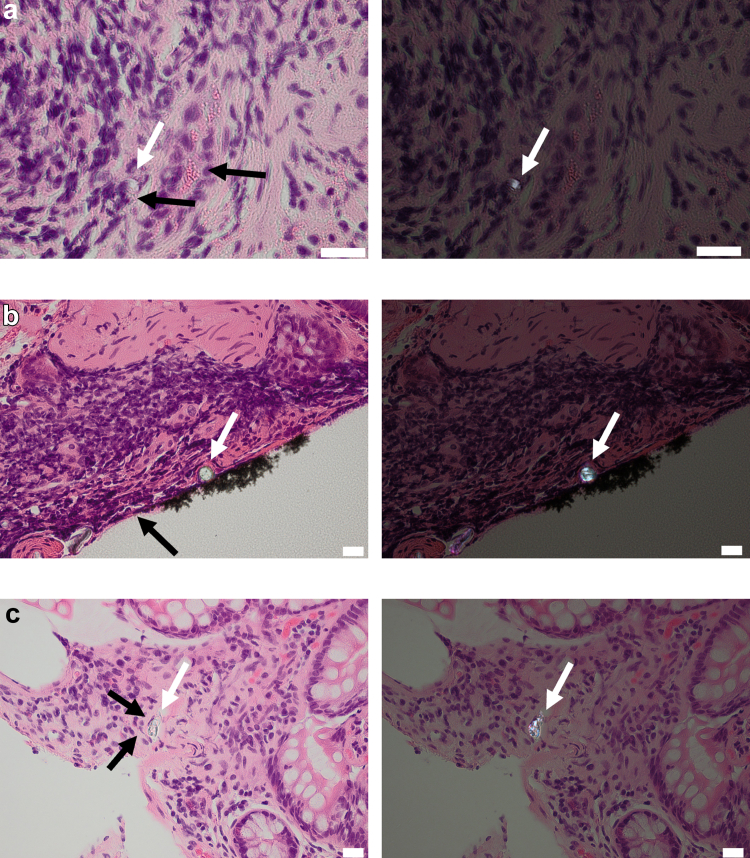
Example micrographs from three different cases where the observed particle did not pass the three tiers of the suspect screen (out of 12 at Tier 2) due to a) proximity to a cluster of red blood cells, the presence of tissue overlaid on the particle; b) being close to the edge of the specimen/tape contamination; and c) presence of distorted tissue/cell structures surrounding the particle. White arrows indicate the particle of interest, whilst black arrows illustrate justification for exclusion. White scale bars = 20 μm.

A multi-tiered screening framework was developed and applied to ileal H&E tissue sections, to discriminate the presence of exogenous particles due to exposure (as opposed to procedural artefact), thereby illustrating uptake in the GI mucosa and providing justification for future studies on particles and specifically microplastics in such tissues. Tier 1 of the framework—a PLM screen looking for the presence of birefringent particles—found sub-epithelial particles in tissue sections from 35 of the 101 subjects (35%). After applying inclusion and exclusion criteria to eliminate samples containing particles that are likely procedural artefacts in tier 2 of the framework, 12 subjects remained (Fig. 1). After tier 3, scrutiny by a pathologist, it was concluded that tissue sections from only 2 subjects (1 per subject) contained suspicious birefringent particles which could not be confidently attributed to procedural contamination (Figs. 1 and 2a, b), and one section from one subject contained apparent starch particles,^28^ which was noted of interest due to their relatively large size (Fig. 2c). Thus, particles observed in tissue sections from 32 subjects (i.e., 91%) were considered procedural contaminants, with examples presented in Fig. 3. These include instances where the observed particle was found in proximity to a cluster of red blood cells (Fig. 3a), and thus could be a result of intrusion, or where the tissue overlays the particle, suggesting layering on top of procedural contamination (Fig. 3b). Other examples include when the particle is close to the edge of the specimen, for which contamination of the tape used in specimen processing could not be ruled out, or where the tissue surrounding the particle appears distorted as if forced in during processing. These results are summarised in Table 3. Raw data tables can be found in Supplementary Materials (Tables S1–S3).

**Table 3:** Summary of the number of cases (subjects with ileal tissue sections containing sub-mucosal exogenous particles) remaining at the start and end of each tier following the *in situ* Particle Screening Framework.

	Start	End
Tier 1	101	35
Tier 2	35	12
Tier 3	12	3

The particles observed in Fig. 2a and b were 16.67 and 19.69 μm, respectively, whilst the starch particles (Fig. 2c) were on average 29.1 μm (Feret maximum). It took approximately two weeks to screen 164 slides from 101 subjects and apply the different tiers of evaluation to decreasing slide numbers. The frequency of clearly identified submucosal birefringent particles (excluding starch) was thus low, i.e., in almost 3% of subjects. In terms of consecutive observations within specimen, sub-epithelial birefringent particles were found in 2–3 consecutive tissue sections (4 μm apart) from eight subjects overall, however, these did not pass Tier 2 of the framework.

**Fig. 4:**
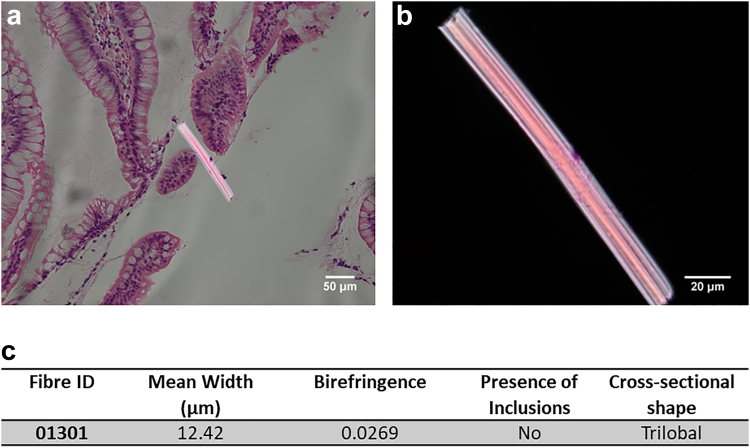
A synthetic fibre in the lumen of an ileum tissue section. a) A micrograph of an ileum tissue section with a Rayon viscose microfibre in the gastrointestinal lumen, with mucus and macrophages adhered to its surface (inset); scale bar = 50 μm; b) the same Rayon viscose microfibre under crossed polars on a polarised light microscope; scale bar = 20 μm; c) and the optical examination results of the microfibre contained in the sample.

During the screen, a suspicious birefringent fibre (158.78 μm ± 0.89 × 12.42 μm ± 1.11) was observed in a tissue section in the current study, in the gastrointestinal lumen (Fig. 4a). Due to its orientation in the section, we were able to observe its cross-section, which was distinctly trilobal (Fig. 4b). With a birefringence value of 0.0269 (Fig. 4c), it was most likely a viscose fibre, often termed Rayon and categorised as a semi-synthetic man-made cellulose fibre. Two cells are present on the surface of the fibre (Fig. 4a); however, due to the limitations of the nature of the samples being H&E slides, we cannot definitively confirm their cell type. Their appearance suggests they might be macrophages, but the observed dimensions are too small for them to be complete cells. Given that the sections are only 3 μm thick, it is likely that we are observing partial sections of two macrophages, each cut through a different plane of the cell body. After 1–2 h incubation with a substrate, macrophages are adherent enough to withstand washing procedures^29^ which suggests that the fibre has been caught in the mucus layer between the small bowel villi for a minimum of 1–2 h to allow the macrophages to migrate from a nearby capillary into the bowel lumen and become adherent to the fibre. As subjects undergoing endoscopy and biopsy were on a low residue diet on the day prior to the procedure, had taken a laxative and had no solid food prior to the biopsy, the identification of this fibre suggests it was persistent within the gut and not simply associated with a recent food bolus.

### Chemical analysis

**Fig. 5:**
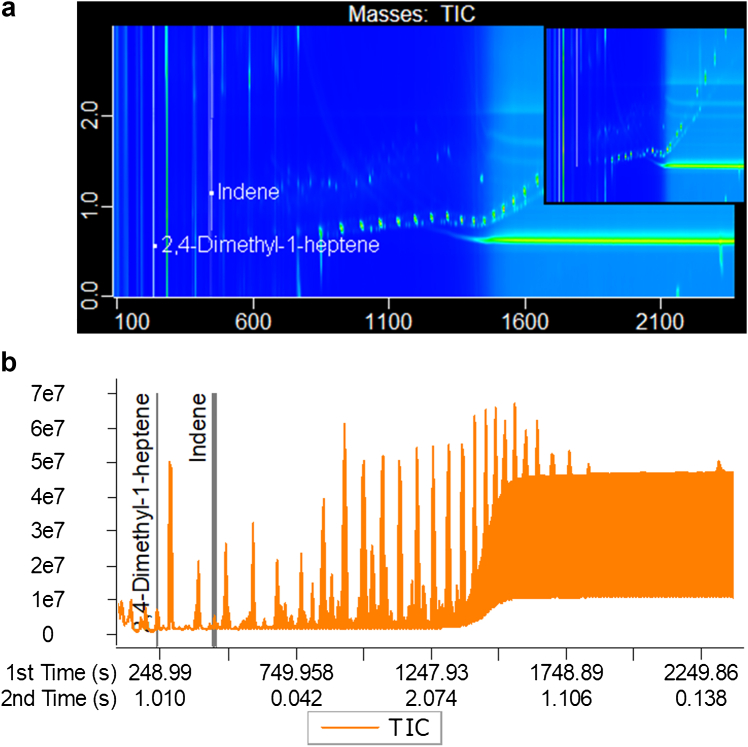
Indicative qualification of the presence of polypropylene and polyvinylchloride polymers in exemplary bulk digested colon tissue (n = 1). a) An annotated 2D total ion chromatogram (TIC) plot, where each axis represents retention time in the 1st and 2nd dimension (primary and secondary gas chromatograph columns, respectively). b) The corresponding pyrogram. Both were generated in ChromaTOF software (Leco).

To demonstrate how the *in situ* Particle Screening Framework sits within a larger pipeline of research and how future work would progress having demonstrated that particles can access this tissue, exemplary chemical data was generated to indicate the presence of plastic in gut tissue. Analysis of bulk gut (colon) tissue sampled separately and for the purpose of plastic analysis found signatures of two of twelve targeted plastic polymers (Fig. 5). The presence of polypropylene (PP) and polyvinylchloride (PVC) was indicated by the detection of their marker ions (indene, naphthalene and fluorene for PVC, and 2,4-dimethyl hept-1-ene for PP), which were not detected in the corresponding procedural blank (Table 4).

**Table 4:** The qualitative presence/absence of the targeted plastic polymers in a bulk digested gut (colon) tissue sample and a procedural blank; “–” indicates presence.

	ABS	N6	N66	PE	PET	PC	PMMA	PP	PU	PVC	SBR
Procedural Blank											
Gut Tissue								–		–	

## Discussion

The aim of the current study was to develop and apply a screening framework which increases the level of confidence in *in situ* particle and microplastic data from pathological tissue sections, acting as a proof of principal in determining whether discriminate foreign microparticles gain access to tissues. To our knowledge, this is the first framework proposed of its kind, although previous publications have explored paraffin-embedded tissue sections for foreign materials, predominantly through elemental analysis of particles.^30^ In one study, a multi-modal micro (spectro)scopy approach was used to identify acrylic polyamide microspheres in formalin fixed paraffin embedded (FFPE) sections from patients in receipt of vascular embolisation treatments.^31^

The approximate reduction of 91% in particle-positive samples following the application of the *in situ* Particle Screening Framework highlights the importance of having various tiers of QAQC checks, such as exclusion criteria and expert opinions. It also emphasises the ubiquity of particulate contamination in the histopathological process, from specimen excision/biopsy through to section processing.^32^ Such contamination could occur due to physical trauma to the tissue during surgery or biopsy, which could artificially introduce plastic material, e.g., from the apparatus, tissue processing workflow or even ambient contamination from the air. Foreign material has historically been observed in histopathology tissue sections.^33^ The current study goes beyond this, by proposing a level of scrutiny to ensure greater confidence in the observation that detected particles are in tissues due to prior environmental exposure.

This is crucial as high impact studies emerge. However, microplastic particles detected are often orders of magnitude larger than what previous research fields have determined plausible.^7^

A key gap in understanding in microplastic research is dosimetry and ADME in humans following exposure. In the gut there is potential for particle translocation via endocytosis at the Peyer's Patches^34^ or persorption at the microvilli,^35^ hence why ileum tissue sections were screened through all tiers in the present study. Birefringent particles were observed in the subepithelial mucosa of three subjects. The presence of relatively large birefringent subepithelial particles including starch particles^28^ in this study is of note, as it supports the theory of persorption. Persorption defines the phenomenon whereby micrometre-sized resistant particles (e.g., starch, mineral dust) are mechanically kneaded across gaps in the intestinal epithelium resulting from cell shedding.^35^ It is thus plausible that microplastic particles may persorb across the epithelium. This would support the observation of larger particles in gastrointestinal tissue, up to a maximum of ∼130 μm, as defined experimentally in humans for non-digestible starch particles,^36^ assuming particle size is the restricting factor. The sizes of particles in the subepithelial mucosa in the present study overlap with microplastic particles commonly encountered in the environment and in biological samples (e.g.,^37^^,^^38^), although this alone should not be used as a credibility check. That is why the screen was developed and performed in this study: 1) to exploit clues in the surrounding tissue/section to distinguish true and false positives, and 2) to understand the plausibility of microparticles being incorporated into the mucosa.

**Fig. 6:**
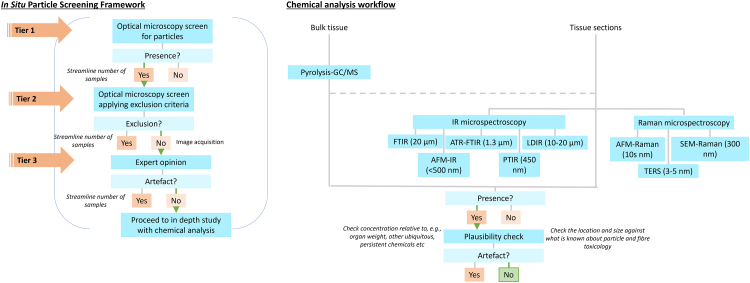
A schematic overview of the *in situ* Particle Screening Framework proposed for microplastic research in the context of the recommended analytical pipeline.

Following the broader proposed framework (Fig. 6), a next step would be to design follow-up studies which further analyse particles using a technique which can classify them based on their chemical composition, ideally using structural information as opposed to elemental data alone. For H and E-stained histology slides, this is a challenge, as components such as the formalin in which the specimen is fixed, paraffin in which the specimen is embedded, glass cover slips and slides, and the various stains used on the tissue will each have their own active signal or fluorescence for the various techniques often used to detect and identify microplastic particles, notably infrared (IR) and Raman vibrational microspectroscopy.

Alternatively, bulk tissue could be analysed using Py-GCMS to indicate the presence of plastic within a sample. We have included example chemical data from the human gut (specifically colon) using Py-GCxGC TOFMS, a variation of Py-GCMS. This tissue was accessible and available fresh-frozen, which is optimum for the subsequent processing and analysis. Moreover, colon contains similar lymph tissues. Signatures of plastic were observed in human colon tissue, further demonstrating how this research pipeline would progress. The observation of the fibre in the lumen suggests that, in some cases, microplastic particles recovered from digested gastrointestinal tissue may have only been in the overlying lumen as opposed to embedded in underlying tissue. This could explain why some studies observe and report particles of a size too large to have crossed the gastrointestinal barrier.^39^^,^^40^

H and E slides and FFPE specimen present a vast resource. Some options to make this useable for these purposes include lifting the cover slip prior to microspectroscopy to remove potential interference from glass or deducting a glass background from the sample spectrum (not necessary for confocal Raman, which can focus through the layer of glass), applying a decolourisation step to minimise interference from the stains, and deparaffinising the sections to remove the paraffin signal. The effect of all of these on plastic particles must be systematically evaluated before application. All these steps also present an opportunity for contamination, but these microplastic particles would be easy to distinguish during screening (second tier) as they would contaminate the section surface in a different focal plane. Ideally, a method with the minimum number of processing steps is preferred to avoid any chance of introducing artefacts leading to false positive results and for efficiency. An alternative approach could be to analyse unstained, uncovered sections first, before staining after chemical analysis. Should this be followed, exclusion criteria 2, 3, and 5 of the framework can be applied. Post-staining, criteria 1 and 4 can be checked. An optimal workflow for microspectroscopy would be to use unstained frozen tissue, to which Tier 1 and Tier 2 Criteria 2, 3, and 5 are applied prior to chemical analysis. After chemical analysis, the section is stained and the remaining criteria and then Tier 3 are followed. This would clarify the tissue architecture and local cellular response to help determine whether observed microplastic particles are overlaid on top, underneath or pressed into the top of the section due to procedural contamination from slide preparation. This all depends on the sample preparation, which should be designed into future studies which include chemical characterisation, having initially demonstrated that suspect exogenous particles access target tissues.

A limitation of the current study is that the acquired ethics did not cover demographic information about the sample population. This was because the study is about proof of concept that particles gain access to tissues using a developed screening framework. Future work could retrospectively assess the relationship of particle prevalence in archived tissues with demographic factors such as age, gender, or comorbidities, e.g., which lead to leaky epithelial barriers, alongside case controls, to identify vulnerable populations. Additionally, specific pathologies could be targeted in archived tissues following a case–control design, to explore associations between exogenous particles and disease. An additional limitation was that the PLM screen was performed manually. Future methodological improvements could adapt the screening to higher throughput microscopy such as automated imaging. Strengths of the current study include a verified methodology for distinguishing particles in tissues and using the histopathological context to rule out artefacts. This will be useful for researchers interested in screening tissue libraries for evidence of particle uptake and for identifying target tissue for future research on associated pathologies. An additional strength is that the findings contribute evidence on the presence of microparticles in the gastrointestinal mucosa, supporting a potential mechanism for their uptake and highlighting this tissue as a potential target for particle-related effects.

The emerging field of microplastic research presents unique challenges due to the novel nature of these contaminants and the limitations of current detection and analysis techniques. In this paper we have provided a framework for the robust evaluation of the presence of particles in tissues, that is amendable to tissue archives, and throughout emphasises the need for expert input on the histopathological context (Fig. 6). The key issue throughout is not only the robustness of the metrological approach to the identification of particles including microplastics within human tissues, but also the credibility of the observation within the context of what is already known about particle and fibre uptake, distribution and accumulation within the human body. Whilst microplastic research is an emerging field, particle and fibre toxicology is not, and there is much to be learnt, and cautionary lessons to be applied by relating new observations to this extensive evidence base. To establish whether microplastic identifications in tissues are credible we propose the adoption of two of the nine Bradford Hill criteria, ‘plausibility’ and ‘analogy’.^41^ Whilst the application of these criteria is more often applied relating exposures and adverse health effects, to establish the strength of evidence for a cause-and-effect relationship, we believe they can be applied effectively to testing the validity of microplastic identification in tissues. In this context, ‘plausibility’ refers to the extent to which the proposed observation is consistent with existing scientific knowledge concerning particle uptake and distribution in the body. In the case of microplastics, it is important to consider the physical properties of these particles, such as size, shape, and chemical composition, and how these properties may affect their ability to penetrate tissues and interact with cells. ‘Analogy’ here refers to the extent to which the observed distribution and accumulation of microplastics are similar to the patterns observed for other particle and fibre types. Where observations do not align with these requirements, we believe extreme caution should be applied to their interpretation.

This paper has emphasised the importance of interpreting the presence of microplastic and other exogenous materials in the tissue context. Increasingly due to the analytical challenges and relative scarcity of microplastics in tissues, whole tissue digestion and destructive analytical techniques such as Py-GCMS have been applied,^42^, ^43^, ^44^ such as in the current study. Whilst these techniques, when applied after the size fractionation of digests, offer the possibility of examining nanoscale materials, they do so without the tissue context, and as such are not easily evaluated using the scheme above. We therefore believe that these observations should be considered nominal pending confirmation by other imaging methods. Throughout, the questions asked when addressing the plausibility of an observation are very simplistic: is it credible the particles/fibres are within tissues, and if so, are the concentrations reported plausible (relative to other exogenous particle/fibres)? We propose that these considerations are an essential part of any evaluation framework.

## Contributors

SW conceived the study with input from JS. JS coordinated the ethics application. SW performed the tissue screen and wrote the manuscript. JS provided expert pathology guidance and contributed to the manuscript. CG performed fibre identification and contributed to the manuscript. JK provided tissues and provided input to the manuscript. YI processed and analysed samples and provided input to the manuscript. HB analysed, processed and converted chemical data and provided input to the manuscript. IM and FK accessed and verified the screen data generated by SW, whilst SW and JS scrutinised resulting images. FJK and IM reviewed early drafts of the manuscript and provided input to the manuscript. All authors read and approved the final manuscript.

## Data sharing statement

Data will be made available upon request to the corresponding author.

## Declaration of interests

SW has received funding in the past 36 months from the Medical Research Council, Natural Environment Research Council, National Institute for Health Research (NIHR), and the Minderoo Foundation. SW has received travel support from the American Chemistry Council and Minderoo to attend workshops. SW has been involved in a sub-group of the Department of Health and Social Care Committee on the Medical Effects of Air Pollutants (COMEAP) evaluating microplastics. IM is a member (unpaid advisory role) of COMEAP and is also involved in the sub-group evaluating Microplastics. FK is a trustee for Global Action Plan and a board member for Breathe Cities. JK has received grants from NIHR, UKRI, EPSRC and CRUK. JK has received consulting fees from Johnson & Johnson payments/honoraria for presentations from Mindray and Yakult, support for meeting travel from Intuitive and Mindray, is on the safety board for Safeheal, has leadership roles in the Simpson Smith Charity and Bowel Research UK, and is a shareholder in SurgEase Innovations Ltd, Medical iSight, Concentric, The Evidence Company, Intus Biosciences, and Wype Ltd.
