# Supplementary material for: A new screening framework to support the identification of exogenous particles and suspect microplastics in situ in pathological tissue samples
Source: eBioMedicine. 2025 Nov 3;121:105984. doi: 10.1016/j.ebiom.2025.105984 (PMC12629922; doi:10.1016/j.ebiom.2025.105984)
Supplement: Supplementary Tables [file mmc1.docx]

**A new screening framework to support the identification of exogenous particles and suspect microplastics *in situ* in pathological tissue samples**

**Supplementary Information**

Stephanie L Wright^1, 2*^, Claire Gwinnett^3^, Ian Mudway^1, 2^, Yukari Ishikawa^1, 2^, Henry Blake^1, 2^, James Kinross^4^, Frank J Kelly^1, 2^ and Jon Salisbury^4^

^1^ Environmental Research Group, School of Public Health, Imperial College London

^2^ Medical Research Council Centre for Environment and Health, School of Public Health, Imperial College London

^3^ Criminal Justice and Forensic Science Department, School of Law, Policing and Forensic Science, Staffordshire University, Stoke-on-Trent, ST42DF, United Kingdom.

^4^ Department of Surgery & Cancer, Imperial College London

^4^ Department of Histopathology, King’s College Hospital, London SE5 9RS, United Kingdom.

^*^s.wright19@imperial.ac.uk

Table S1. Results from the Tier 1 screen.

| **Subject #** | **Slide #** | **Birefringent particles?** | **Pass Tier 1?** |
| --- | --- | --- | --- |
| 1 | 1 | Y | N |
| 1 | 2 | N | N |
| 1 | 3 | Y | N |
| 2 | 1 | Y | Y |
| 3 | 1 | Y | Y |
| 4 | 1 | Y | Y |
| 4 | 2 | Y | N |
| 4 | 3 | Y | N |
| 5 | 1 | N | N |
| 5 | 2 | N | N |
| 5 | 3 | N | N |
| 6 | 1 | Y | Y |
| 6 | 2 | Y | Y |
| 7 | 1 | Y | Y |
| 8 | 1 | Y | Y |
| 9 | 1 | N | N |
| 9 | 2 | N | N |
| 10 | 1 | Y | Y |
| 11 | 1 | Y | N |
| 12 | 1 | Y | Y |
| 13 | 1 | Y | N |
| 13 | 2 | Y | N |
| 13 | 3 | Y | N |
| 14 | 1 | N | N |
| 14 | 2 | N | N |
| 14 | 3 | N | N |
| 15 | 1 | N | N |
| 16 | 1 | Y | Y |
| 17 | 1 | Y | N |
| 17 | 2 | N | N |
| 18 | 1 | N | N |
| 18 | 2 | Y | N |
| 18 | 3 | N | N |
| 19 | 1 | Y | Y |
| 20 | 1 | Y | N |
| 21 | 1 | Y | N |
| 22 | 1 | N | N |
| 22 | 2 | N | N |
| 23 | 1 | N | N |
| 23 | 2 | N | N |
| 23 | 3 | N | N |
| 24 | 1 | Y | Y |
| 24 | 2 | N | N |
| 25 | 1 | Y | Y |
| 26 | 1 | N | N |
| 26 | 2 | N | N |
| 26 | 3 | N | N |
| 27 | 1 | Y | Y |
| 28 | 1 | N | N |
| 28 | 2 | N | N |
| 28 | 3 | N | N |
| 29 | 1 | N | N |
| 29 | 2 | N | N |
| 30 | 1 | N | N |
| 30 | 2 | N | N |
| 30 | 3 | N | N |
| 31 | 1 | Y | N |
| 31 | 2 | Y | N |
| 31 | 3 | N | N |
| 32 | 1 | Y | N |
| 32 | 2 | Y | N |
| 33 | 1 | Y | Y |
| 33 | 2 | Y | N |
| 33 | 3 | N | N |
| 34 | 1 | Y | Y |
| 35 | 1 | Y | Y |
| 36 | 1 | N | N |
| 37 | 1 | N | N |
| 38 | 1 | Y | N |
| 39 | 1 | N | N |
| 40 | 1 | Y | Y |
| 40 | 2 | Y | N |
| 40 | 3 | N | N |
| 41 | 1 | Y | Y |
| 42 | 1 | N | N |
| 42 | 2 | N | N |
| 42 | 3 | N | N |
| 42 | 4 | N | N |
| 43 | 1 | Y | Y |
| 44 | 1 | N | N |
| 45 | 1 | Y | Y |
| 45 | 2 | Y | Y |
| 45 | 3 | Y | Y |
| 45 | 4 | Y | Y |
| 45 | 5 | Y | Y |
| 45 | 6 | Y | Y |
| 45 | 7 | N | N |
| 46 | 1 | Y | Y |
| 47 | 1 | Y | Y |
| 48 | 1 | N | N |
| 49 | 1 | Y | N |
| 50 | 1 | Y | N |
| 51 | 1 | Y | N |
| 52 | 1 | Y | Y |
| 52 | 2 | Y | Y |
| 53 | 1 | N | N |
| 53 | 2 | Y | N |
| 53 | 3 | Y | N |
| 54 | 1 | Y | N |
| 54 | 2 | Y | N |
| 54 | 3 | Y | N |
| 55 | 1 | Y | Y |
| 56 | 1 | Y | N |
| 57 | 1 | Y | N |
| 57 | 2 | Y | N |
| 58 | 1 | Y | Y |
| 58 | 2 | N | N |
| 58 | 3 | N | N |
| 59 | 1 | Y | N |
| 59 | 2 | Y | N |
| 59 | 3 | Y | N |
| 60 | 1 | Y | N |
| 60 | 2 | Y | N |
| 61 | 1 | Y | N |
| 62 | 1 | N | N |
| 62 | 2 | N | N |
| 62 | 3 | N | N |
| 63 | 1 | Y | Y |
| 64 | 1 | Y | N |
| 65 | 1 | Y | N |
| 66 | 1 | Y | N |
| 67 | 1 | Y | Y |
| 68 | 1 | N | N |
| 68 | 2 | N | N |
| 68 | 3 | N | N |
| 69 | 1 | N | N |
| 69 | 2 | N | N |
| 70 | 1 | N | N |
| 70 | 2 | N | N |
| 70 | 3 | Y | N |
| 71 | 1 | Y | N |
| 72 | 1 | Y | N |
| 73 | 1 | Y | Y |
| 74 | 1 | Y | Y |
| 75 | 1 | Y | Y |
| 75 | 2 | N | N |
| 76 | 1 | Y | N |
| 77 | 1 | Y | Y |
| 78 | 1 | Y | Y |
| 79 | 1 | Y | N |
| 80 | 1 | Y | N |
| 80 | 2 | Y | N |
| 81 | 1 | Y | N |
| 82 | 1 | Y | Y |
| 82 | 2 | Y | Y |
| 83 | 1 | Y | N |
| 84 | 1 | N | N |
| 85 | 1 | Y | Y |
| 85 | 2 | N | N |
| 85 | 3 | N | N |
| 86 | 1 | Y | N |
| 87 | 1 | N | N |
| 88 | 1 | N | N |
| 89 | 1 | N | N |
| 89 | 2 | N | N |
| 90 | 1 | N | N |
| 91 | 1 | Y | N |
| 92 | 1 | Y | N |
| 93 | 1 | Y | N |
| 94 | 1 | N | N |
| 95 | 1 | Y | N |
| 96 | 1 | Y | N |
| 97 | 1 | Y | N |
| 98 | 1 | Y | N |
| 99 | 1 | Y | Y |
| 100 | 1 | Y | N |
| 101 | 1 | Y | N |
| TOTAL |  |  | 35 subjects |

Table S2. Results from the Tier 2 Screen

| **Subject #** | **Slide #** | **Pass Tier 2?** |
| --- | --- | --- |
| 2 | 1 | N |
| 3 | 1 | N |
| 4 | 1 | N |
| 6 | 1 | Y |
| 6 | 2 | N |
| 7 | 1 | N |
| 8 | 1 | N |
| 10 | 1 | N |
| 12 | 1 | Y |
| 16 | 1 | N |
| 19 | 1 | N |
| 24 | 1 | N |
| 25 | 1 | N |
| 27 | 1 | N |
| 33 | 1 | Y |
| 34 | 1 | N |
| 35 | 1 | N |
| 40 | 1 | Y |
| 41 | 1 | N |
| 43 | 1 | Y |
| 45 | 1 | Y |
| 45 | 2 | Y |
| 45 | 3 | Y |
| 45 | 4 | Y |
| 45 | 5 | Y |
| 45 | 6 | Y |
| 46 | 1 | Y |
| 47 | 1 | N |
| 52 | 1 | Y |
| 52 | 2 | Y |
| 55 | 1 | Y |
| 58 | 1 | N |
| 63 | 1 | Y |
| 67 | 1 | N |
| 73 | 1 | N |
| 74 | 1 | N |
| 75 | 1 | N |
| 77 | 1 | N |
| 78 | 1 | Y |
| 82 | 1 | Y |
| 82 | 2 | Y |
| 85 | 1 | N |
| 99 | 1 | N |
| TOTAL |  | 12 subjects |

Table S3. Results from the Tier 3 screen.

| **Subject #** | **Slide #** | **Pass Tier 3?** |
| --- | --- | --- |
| 6 | 1 | N |
| 12 | 1 | N |
| 33 | 1 | Y |
| 40 | 1 | Y |
| 43 | 1 | N |
| 45 | 1 | Y |
| 45 | 2 | Y |
| 45 | 3 | Y |
| 45 | 4 | Y |
| 45 | 5 | Y |
| 45 | 6 | Y |
| 46 | 1 | N |
| 52 | 1 | N |
| 52 | 2 | N |
| 55 | 1 | N |
| 63 | 1 | N |
| 78 | 1 | N |
| 82 | 1 | N |
| 82 | 2 | N |
| TOTAL |  | 3 subjects |
|  |  | (subject #45 = starch) |
